# Prevalence and factors associated with sexual violence against adolescent girls in Brazil: results of the 2019 National Survey of School Health

**DOI:** 10.1590/1980-549720260016

**Published:** 2026-04-03

**Authors:** Maria Luiza Sady Prates Pinheiro, Alanna Gomes da Silva, Nádia Machado de Vasconcelos, Deborah Carvalho Malta

**Affiliations:** I Universidade Federal de Minas Gerais, School of Nursing. Department of Maternal and Child Nursing and Public Health - Belo Horizonte (MG), Brazil.; II Universidade Federal de Minas Gerais, School of Medicine. Department of Preventive and Social Medicine - Belo Horizonte (MG), Brazil.; III Universidade Federal de Minas Gerais, Graduate Program in Public Health - Belo Horizonte (MG), Brazil.

**Keywords:** Epidemiology, Adolescent health, Epidemiological surveys, Brazil, Sexual offenses

## Abstract

**Objective:**

To analyze the prevalence of sexual violence and associated factors among adolescent girls aged 13 to 17 years in Brazil, considering individual, relational, and community aspects.

**Methods:**

This is a cross-sectional study using data from the 2019 National Survey of School Health (PeNSE). Descriptive analyses were conducted and prevalence ratios with 95% confidence intervals were calculated.

**Results:**

The prevalence of sexual violence was 22.2% (95%CI 21.5-23.0), with the main perpetrator being a stranger (22.5%; 95%CI 21.2-23.8). Higher prevalence was observed among adolescents aged 16-17 years (PR 1.2; 95%CI 1.1-1.3), those who used drugs (PR 1.3; 95%CI 1.1-1.4), consumed alcohol (PR 1.2; 95%CI 1.1-1.3), had a history of sexual intercourse (PR 1.5; 95%CI 1.4-1.7), reported sadness (PR 1.9; 95%CI 1.4-2.5), had mothers with Higher Education degree (PR 1.2; 95%CI 1.1-1.3), were bullied (PR 1.2; 95%CI 1.2-1.3), were assaulted by friends (PR 1.2; 95%CI 1.1-1.4), and felt unsafe getting to and from school (PR 1.2; 95%CI 1.1-1.4) or at school (PR 1.3; 95%CI 1.1-1.4).

**Conclusion:**

We verified high prevalence of sexual violence among girls aged 16-17 years in Brazil. It was associated with individual, social, and community factors. Multifaceted interventions, strengthening of intersectoral public policies, and protective strategies are needed to ensure a safer environment.

## INTRODUCTION

Sexual violence (SV) against adolescent girls is a serious violation of human rights and a persistent public health issue^1^. It consists in any sexual-related act, whether carried out or attempted, including sexual exploitation, that causes physical, psychological, or social damage, as well as conducts that force the victim to witness or practice sexual acts, in a face-to-face or virtual way^2^ ^,^ ^3^. This form of violence is prevalent in this age group^2^ ^,^ ^4^ ^,^ ^5^, affecting the development, self-esteem, mental health, and school performance of adolescents^6^.

Violence is a complex phenomenon related to cultural, social, and economic factors^7^ ^,^ ^8^, especially affecting vulnerable groups such as children, adolescents, girls, and women^9^ ^,^ ^10^. In this context, SV against adolescent girls reflects a structural dynamic of inequalities and violations, in addition to a patriarchal society that supports male domination in several spheres^11^ ^,^ ^12^ ^,^ ^13^.

Adolescence is marked by biological, psychological, and emotional changes essential to human development^7^ ^,^ ^14^. In this stage, the family context has a great influence on the well-being of adolescents, making them more vulnerable to the physical and social dynamics of the environment^15^. In situations of social, school, and economic vulnerability, these factors intensify, increasing the risk of exposure to violence and other health issues^16^ ^,^ ^17^.

Violence is one of the main causes of morbidity and mortality among adolescents in Brazil^18^, with lasting impacts on various areas of life^7^ ^,^ ^14^ ^,^ ^19^. It is associated with increased risk of mental disorders, use of alcohol, drugs, tobacco, and unprotected sexual practices^20^. It also increases exposure to chronic diseases, such as cancer, diabetes, and cardiovascular diseases, as well as infections such as HIV^19^.

Globally, it is estimated that 370 million girls and women have been raped or sexually abused during childhood^21^. From 2015 to 2021, 92.7% of suspected or confirmed SV notifications concerned adolescent girls^22^. In 2022, 87.7% of victims of SV under 19 years of age were girls^23^, and in 2023, 49.6% of the assaults recorded against adolescents aged 10 to 14 years were of sexual nature^2^. Still in 2023, rape cases increased by 1.54% compared to the previous year, with the majority of victims being women^24^.

Due to the impact and magnitude of gender violence, Brazil is committed to the 2030 Agenda of the Sustainable Development Goals (SDGs), whose aim is to achieve gender equality and empower women and girls, in addition to eliminating gender violence^25^.

In Brazil, studies whose authors comprehensively investigate factors associated with SV in adolescent girls are still limited based on national population data. Most research focuses on local samples encompassing both sexes^26^ ^,^ ^27^ ^,^ ^28^ ^,^ ^29^ or addresses violence in general^4^ ^,^ ^8^ ^,^ ^11^ ^,^ ^30^ ^,^ ^31^. Despite advancing in understanding the vulnerability and risk factors of sexual violence, these studies have methodological limitations — such as small samples, regional focus, and lack of analysis by sexes. Furthermore, relational and community dimensions are approached by a few in an integrated way. By using data from the 2019 National Survey of School Health (*Pesquisa Nacional de Saúde do Escolar* - PeNSE), in this analysis, we contribute to increasing the knowledge of SV among girls on a national scale, offering subsidies for more effective public policies. Unlike studies on the 2015 PeNSE edition^27^ ^,^ ^32^, which presented data aggregated by sex, the 2019 one allows an analysis more sensitive to gender inequalities.

Therefore, our objective was to analyze the prevalence of SV and associated factors among adolescent girls aged 13 to 17 years in Brazil, considering individual, relational, and community aspects.

## METHODS

This is a cross-sectional and analytical study, with data from 2019 PeNSE, carried out by the Brazilian Institute of Geography and Statistics (IBGE) in partnership with the Brazilian Ministry of Health^33^.

The sampling plan of the research was constituted by clusters in two stages: schools and classes of enrolled students, and considered the following geographic levels: Brazil; major regions; Federative Units; capital cities; and the Federal District^33^.

Data were collected by a self-administered structured questionnaire, completed by the students on a smartphone. The weights of schools, classes, and students were adjusted based on data of the School Census. In 2019, 125,123 questionnaires were analyzed, collected in 4,242 schools and 6,612 classes, with participation of 189,857 enrolled students and 183,264 regular attendees^33^.

PeNSE participants are adolescents aged 13 to 17 years, enrolled and attendees, from the 7th to 9th grades of Elementary School and from the 1st to 3rd grades of High School in public and private schools in Brazil^33^. For this study, the population of adolescent girls, aged 13 to 17 years, who had their questionnaires valid (n=63,148), were considered.

The dependent variable of the study was SV, being created from the indicators present in the “Safety” section of the questionnaire, referring to safety situations in the environment in which the student lives. The SV variable is composed of adolescent girls who answered “yes” to one of the following indicators^26^:

1. Sexual abuse: Prevalence of school-age girls who have ever been touched by someone, manipulated, kissed, or who have suffered exposure of body parts against their will;
2. Rape: Prevalence of school-age girls who have ever been threatened by someone, intimidated, or forced to have sex or any other sexual act against their will.

For girls who answered “yes” to one of the indicators, associated variables were also analyzed, namely: age of the first rape and perpetrator’s identity. Age was investigated by the question: “What age were you when someone threatened, intimidated, or forced you to have sex or any other sexual act against your will for the first time?” (9 years or younger; 10 to 13 years; 14 to 15 years; and 16 to 17 years). In turn, the perpetrator’s identity was classified in the following categories: boyfriend, ex-boyfriend, casual date, crush; friend; father, mother, stepfather or stepmother, other relatives, stranger, and others.

The independent variables related to SV were created based on the ecological model of violence, as proposed by Dahlberg and Krug^34^, which, in turn, was adapted from the ecological model of human development by Bronfenbrenner^35^. The model evaluates factors related to violence at four levels: individual, relationships, community, and society. The first level is related to individual characteristics; the second, to close social relations; the third, to community contexts in which the individual is inserted; and the fourth, to broader social factors affecting violence rates^34^. In this study, the model was used exclusively as a theoretical reference for the categorization of variables and interpretation of results, without application of statistical techniques of multilevel analysis. Therefore, the variables were divided into three levels: individual, relationships, and community. The fourth level, society, was not evaluated because PeNSE does not cover this aspect.

The independent variables included in the model were described by relative frequencies (%) and the respective 95% confidence intervals (95%CI), based on the categories available in the database. Next, the definition and categorization of these variables are presented, according to the ecological model of violence adopted for the analysis:

• Individual factors (level 1): age group (13 to 15 years; 16 and 17 years); skin color (white, Black, mixed-race and others — Asian and Indigenous); use of illicit drugs — marijuana and crack cocaine (yes; no); alcohol consumption in the last 30 days (yes; no); use of cigarettes in the last 30 days (yes; no); use of other tobacco products in the last 30 days (yes; no); history of sexual activity (yes; no); feeling of sadness (yes; no); and feeling that life is not worth living (yes; no).
• Close social relations (level 2): maternal level of education (illiterate or complete and incomplete Elementary School, complete and incomplete High School, or incomplete or complete Higher Education); parents or guardians who are smokers (yes; no); parents or guardians who consume alcoholic beverages (yes; no); live with their parents (yes; no); have meals with their parents (yes; no); parents understand their issues and concerns (yes; no); have close friends (yes; no); had been involved in fights (yes; no); bullies or is bullied (yes; no); have been assaulted by friends (yes; no); and have helpful friends (yes; no).
• Community contexts (level 3): housing situation (urban and rural); type of school (private and public); safety getting to and from school (yes; no); safety at school (yes; no); and region (Northeast, North, Southeast, South, and Midwest).

Descriptive analyses were carried out by calculating the prevalence and respective 95%CI of the dependent and independent variables as well as the prevalence of violence per Federative Unit. Prevalence ratios (PR) were estimated with their respective 95%CI using Poisson regression models with robust variance. Bivariate analyses were performed to obtain the crude PR and 95%CI. Multivariate analysis was carried out, including variables with p≤0.05 in the crude analysis, and the backward method was used to create the multivariate regression model. In the final model, variables with p≤0.05 were considered as associated factors. The structure of the sampling process and the post-stratification weights were considered in the analysis of the results. The Software for Statistics and Data Science (Stata), version 14.0, was used through the survey module, which considers the effects of the sampling plan.

The 2019 PeNSE complies with the Guidelines and Regulatory Standards for Research involving Human Beings and was approved by the National Research Ethics Committee of the Ministry of Health, under Opinion No. 3.249.268 on April 8, 2019. Students were informed about the research, their free participation, and that they could withdraw their participation if they did not feel comfortable answering the questions.

## Data Availability Statement:

The entire dataset that supports the results of this study was published in the very article.

## RESULTS

Sexual abuse was reported by 20.2% (95%CI 19.5-21) of adolescents; rape, by 8.9% (95%CI 8.4-9.3); and SV, defined as the occurrence of sexual abuse and/or rape, was reported by 22.2% (95%CI 21.5-23) of these adolescents. Co-occurrence of sexual abuse and rape was observed in 7.0% (95%CI 6.6-7.4) of the participants. The main perpetrators of SV were strangers (22.5%; 95%CI 21.2-23.8), followed by a family member (19.1%; 95%CI 17.7-20.5), boyfriend (19.0%; 95%CI 17.7-20.4), friend (16.9%; 95%CI 15.7-18.2), others (16.1%; 95%CI 14.8-17.6), and parents (6.6%; 95%CI 5.8-7.5).

Regarding individual factors, 27.1% (95%CI 26.0-28.3) of adolescents who reported SV aged 16 to 17 years. However, 37.3% stated that the first episode of violence occurred when they were 10 to 13 years old — 30.4% aged 9 years or younger; 24.5% aged 14 to 15 years; and 7.9% aged 16 to 17 years. SV was more prevalent among girls who reported illicit drug use (PR 46.2%; 95%CI 42.4-50.1); consumed alcoholic beverages in the last 30 days (PR 31.6%; 95%CI 30.2-33.1); smoked cigarettes in the last 30 days (PR 40.7%; 95%CI 36.6-44.8); and reported having a history of sexual activity (PR 34.0%; 95%CI 32.7-35.3), as shown in Table 1.

**Table 1.** Characteristics and crude prevalence ratio of sexual violence among adolescent girls who suffered sexual violence. National Survey of School Health, 2019.

Variables	% (95%CI)	cPR (95%CI)	p-value
Individual factors			
Age group (years)			
13 to 15	19.6 (18.7-20.5)	*	<0.001
16 and 17	27.1 (26.0-28.3)	1.4 (1.3-1.5)	
Skin color			
White	23.8 (22.6-25)	*	<0.001
Black	21.8 (19.9-23.8)	0.9 (0.8-1)	
Mixed-race	20.9 (20-21.9)	0.9 (0.8-0.9)	
Others	24.4 (22-27)	1.0 (0.9-1.1)	
Use of illicit drugs			
No	21.0 (20.3-21.8)	*	<0.001
Yes	46.2 (42.4-50.1)	2.2 (2-2.4)	
Consumption of alcoholic beverages in the last 30 days			
No	18.2 (17.5-18.9)	*	<0.001
Yes	31.6 (30.2-33.1)	1.7 (1.6-1.8)	
Use of cigarettes in the last 30 days			
No	21.0 (20.2-21.7)	*	<0.001
Yes	40.7 (36.6-44.8)	1.9 (1.7-2.2)	
Use of other tobacco products in the last 30 days			
No	20.3 (19.5-21.1)	*	<0.001
Yes	36.8 (34.2-39.4)	1.8 (1.7-2)	
History of sexual activity			
No	17.0 (16.2-17.8)	*	<0.001
Yes	34.0 (32.7-35.3)	2.0 (1.9-2.1)	
Feeling of sadness			
No	7.8 (6-10)	*	<0.001
Yes	23.0 (22.3-23.8)	3.0 (2.3-3.8)	
Feeling that life is not worth living			
No	12.8 (11.8-13.8)	*	<0.001
Yes	27.4 (26.4-28.3)	2.1 (2-2.3)	
Close social relations			
Maternal education			
Illiterate and c/i Elementary School	21.2 (19.9-22.5)	*	<0.001
c/i High School and incomplete Higher Education	24.1 (22.9-25.3)	1.1 (1-1.2)	
Complete Higher Education	25.8 (24.3-27.3)	1.2 (1.1-1.3)	
Parents or guardians who are smokers			
No	21.2 (20.4-22.1)	*	<0.001
Yes	24.8 (23.4-26.2)	1.2 (1.1-1.2)	
Parents or guardians who consume alcoholic beverages			
No	19.0 (17.9-20.1)	*	<0.001
Yes	24.2 (23.2-25.2)	1.3 (1.2-1.4)	
Live with their parents			
No	26.8 (24.6-29.2)	*	<0.001
Yes	21.9 (21.1-22.7)	0.8 (0.7-0.9)	
Have meals with their parents			
No	34.4 (31.5-37.5)	*	<0.001
Yes	21.3 (20.5-22.1)	0.6 (0.6-0.7)	
Parents understand their issues and concerns			
No	31.3 (29.6-33.1)	*	<0.001
Yes	20.2 (19.4-21)	0.6 (0.6-0.7)	
Have close friends			
No	30.5 (26.7-34.5)	*	<0.001
Yes	21.9 (21.2-22.7)	0.7 (0.6-0.8)	
Involvement in fights			
No	21.3 (20.5-22.1)	*	<0.001
Yes	35.8 (33.1-38.6)	1.7 (1.5-1.8)	
Bullying practices			
No	21.1 (20.4-21.9)	*	<0.001
Yes	33.1 (30.8-35.6)	1.6 (1.5-1.7)	
Have been bullied			
No	17.4 (16.6-18.2)	*	<0.001
Yes	28.6 (27.4-29.8)	1.6 (1.6-1.7)	
Have been assaulted by friends			
No	20.7 (19.9-21.4)	*	<0.001
Yes	34.4 (32-36.9)	1.7 (1.5-1.8)	
Have helpful friends			
No	22.1 (19.4-25.2)	*	0.090
Yes	22.3 (21.5-23)	1.0 (0.9-1.1)	
Community contexts			
Housing situation			
Urban	22.7 (21.9-23.4)	*	0.001
Rural	17.3 (14.5-20.5)	0.8 (0.6-0.9)	
Type of school			
Private	24.9 (24-25.8)	*	<0.001
Public	21.8 (21-22.7)	0.9 (0.8-0.9)	
Safety getting to and from school			
Yes	20.4 (19.7-21.1)	*	<0.001
No	34.4 (31.7-37.2)	1.7 (1.6-1.8)	
Safety at school			
Yes	20.1 (19.4-20.9)	*	<0.001
No	36.3 (34.0-38.7)	1.8 (1.7-1.9)	
Region			
North	23.2 (21.1-25.5)	*	0.012
Northeast	20.3 (19.4-21.3)	0.9 (0.8-1)	
Southeast	23.3 (21.7-24.9)	1.0 (0.9-1.1)	
South	22.2 (20.4-24.2)	1.0 (0.8-1.1)	
Midwest	23.0 (21.6-24.5)	1.0 (0.9-1.1)	

95%CI: 95% confidence interval; cPR: crude prevalence ratio; c/i: complete/incomplete.

*Reference variable of prevalence ratio.

As for close social relations, we found a higher prevalence among girls whose parents were smokers (PR 24.8%; 95%CI 23.4-26.2); whose parents consumed alcoholic beverages (PR 24.2%; 95%CI 23.2-25.2); girls who did not live with their parents (PR 26.8%; 95%CI 24.6-29.2); and who did not have meals with their parents (PR 34.4%; 95%CI 31.5-37.5), as demonstrated in Table 1.

Regarding community contexts, we verified higher prevalence among those living in urban areas (PR 22.7%; 95%CI 21.9-23.4); who attended private schools (PR 24.9%; 95%CI 24-25.8); who felt unsafe getting to and from school (PR 34.4%; 95%CI 31.7-37.2) and at school (PR 36.3%; 95%CI 34.0-38.7), according to data in Table 1.

**Graph 1.**
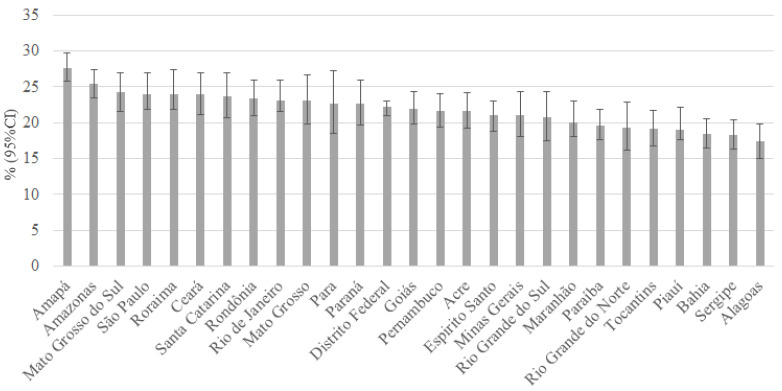
Prevalence of sexual violence in adolescent girls aged 13 to 17 years, according to Federative Units. National Survey of School Health, 2019.

In Graph 1, we show the prevalence of adolescents who suffered SV, according to Federative Units. Although Amapá (27.7%) has the highest prevalence, followed by Amazonas (25.4%) and Mato Grosso do Sul (24.2%), the confidence intervals of the estimates overlap, which implies that the observed differences may not be statistically significant.

In Table 2, we present the final multivariate model. The highest PR of SV was observed among adolescents aged 16 to 17 years (PR 1.17; 95%CI 1.08-1.27); who used drugs (PR 1.27; 95%CI 1.14-1.41); consumed alcohol in the last 30 days (PR 1,18; 95%CI 1.10-1.27); had a history of sexual activity (PR 1.54; 95%CI 1.42-1.67); who reported sadness (PR: 1.85; 95%CI 1.35-2.54); and who thought that life was not worth living (PR 1.51; 95%CI 1.38-1.66). In addition, having mothers with complete Higher Education (PR 1.21; 95%CI 1.09-1.34), being bullied (PR 1.24; 95%CI 1.16-1.33), having been assaulted by friends (PR 1.24; 95%CI 1.14-1.35), feeling unsafe getting to and from school (PR 1.24; 95%CI 1.14-1.36) and at school (PR 1.25; 95%CI 1.14-1.36) also showed positive associations. Conversely, lower PR of SV were verified among adolescents who had parents who understood their issues and concerns (PR 0.83; 95%CI 0.74-0.93) and among those who attended public schools (PR 0.84; 95%CI 0.78-0.89).

**Table 2.** Multivariate model of sexual violence in adolescent girls aged 13 to 17 years. National Survey of School Health, Brazil, 2019.

Variables	adjPR (95%CI)	p-value
Individual factors		
Age group (years)		
13 to 15	*	<0.001
16 and 17	1.17 (1.08-1.27)	
Skin color		
White	*	
Black	0.88 (0.79-0.98)	0.031
Mixed-race	0.94 (0.87-1.01)	0.121
Others	1.03 (0.92-1.16)	0.542
Use of illicit drugs		
No	*	<0.001
Yes	1.27 (1.14-1.41)	
Consumption of alcoholic beverages in the last 30 days		
No	*	<0.001
Yes	1.18 (1.10-1.27)	
History of sexual activity		
No	*	<0.001
Yes	1.54 (1.42-1.67)	
Feeling of sadness		
No	*	<0.001
Yes	1.85 (1.35-2.54)	
Feeling that life is not worth living		
No	*	<0.001
Yes	1.51 (1.38-1.66)	
Close social relations		
Maternal education		
Illiterate and complete and incomplete Elementary School	*	<0.001
Complete and incomplete High School and incomplete Higher Education	1.14 (1.06-1.22)	
Complete Higher Education	1.21 (1.09-1.34)	
Have meals with their parents		
No	*	0.001
Yes	0.83 (0.74-0.93)	
Parents understand their issues and concerns		
No	*	<0.001
Yes	0.83 (0.74-0.93)	
Bullying practices		
No	*	0.031
Yes	1.10 (1.00-1.19)	
Have been bullied		
No	*	<0.001
Yes	1.24 (1.16-1.33)	
Have been assaulted by friends		
No	*	<0.001
Yes	1.24 (1.14-1.35)	
Community contexts		
Administrative unit of the school		
Private	*	<0.001
Public	0.84 (0.78-0.89)	
Safety getting to and from school		
Yes	*	<0.001
No	1.24 (1.14-1.36)	
Safety at school		
Yes	*	<0.001
No	1.25 (1.14-1.36)	
Region		
North	*	
Northeast	0.88 (0.78-0.98)	0.033
Southeast	0.86 (0.76-0.98)	0.028
South	0.89 (0.77-1.02)	0.107
Midwest	0.90 (0.80-1.02)	0.118

adjPR: adjusted prevalence ratio; 95%CI: 95% confidence interval.

*Reference variable of prevalence ratio.

## DISCUSSION

About 22.2% of Brazilian adolescent girls were victims of SV. Higher PR values were verified among adolescents aged 16 to 17 years; who used illicit drugs and alcohol; have had sexual relations; reported feelings of sadness and that life is not worth living; had mothers with complete Higher Education; were bullied; were assaulted by friends; and who felt unsafe getting to and from school and at school. Conversely, lower PR values were observed among those who attended public schools and whose parents understood their issues and concerns.

The prevalence of SV observed in this study was considerably higher than that found in a survey whose authors used PeNSE data from 2015, which pointed to a rate of 4.3% among girls attending the 9th grade of Elementary School^27^. This discrepancy can be partially explained by relevant methodological differences between the two editions of the survey. In 2015, the question about SV was formulated in a more general way and without exemplifications, which may have limited the acknowledgment of the experience of violence situations by adolescents. In turn, in 2019, in addition to the expansion of the target population (from the 7th grade of Elementary School to the 3rd grade of High School), there was a differentiation between sexual abuse and rape, with more specific and exemplary questions, favoring greater understanding and reporting.

Although the reports of SV were predominant among adolescents aged 16 to 17 years, possibly due to the longer life span and the greater understanding of violence, a higher prevalence of the first episode of SV was observed in childhood and preadolescence, i.e., girls aged 10 to 13 years. Authors of other studies point girls aged 10 to 14 years as the most vulnerable group to SV, often occurring in the domestic environment^2^ ^,^ ^27^.

Overall, it is estimated that one out of five women were victims of SV during childhood^36^. Factors, such as low level of education, limited access to means of protection, and economic dependence, especially when not accompanied by adequate family support, increase the vulnerability of adolescent girls to SV^37^.

Regarding the perpetrators, the main individuals were strangers, followed by family members. However, researchers point to the household as the main scenario of occurrence of this violence, with family members being the main perpetrators^11^ ^,^ ^21^ ^,^ ^38^. This discrepancy may be associated with the greater ability of victims to identify and report episodes of violence occurring outside the family environment, while the naturalization of violence in the domestic context, influenced by sociocultural factors, may hinder its acknowledgment and reporting^39^.

Regarding substance use, adolescents who consumed illicit drugs showed a higher prevalence of SV. At this stage, the search for ways to cope with difficult experiences exposes them to drug use^40^ ^,^ ^41^ ^,^ ^42^. According to studies, the use of this type of substance, both licit and illicit, is a behavior that increases the vulnerability of adolescents, causing changes in their behavior, such as reducing the capacity to perceive the environment, making the adolescent more vulnerable^27^ ^,^ ^28^ ^,^ ^31^ ^,^ ^42^.

Another relevant factor is that girls who have had sexual intercourse had a higher prevalence of SV. Adolescence, marked by discoveries and greater social interaction, includes affective and sexual relations^14^, which can increase exposure to vulnerabilities. Thus, sexually-active adolescents tend to face more risk situations related to SV^43^ ^,^ ^44^. Risky sexual behaviors, such as more partners and greater exposure to sexually transmitted infections, can generate negative impacts on the physical, mental, and psychosocial development, with significant repercussions on adult life^45^.

Moreover, adolescents who presented feelings of sadness and the perception that life is not worth living had a higher prevalence of SV. Exposure to violence in adolescence may be associated with an approximately twice greater risk of developing mood and anxiety disorders^45^. In addition, it may increase suicide attempts, psychiatric hospitalizations, self-harm, mood and behavioral disorders, dissociative symptoms, post-traumatic stress disorder, and somatization^47^.

Adolescents whose mothers had higher levels of education had a higher prevalence of SV. Nevertheless, authors of a study with 2015 PeNSE data identified a higher prevalence of SV among adolescents whose mothers had lower levels of education^27^. Higher levels of education can favor the identification of cases of violence and the acknowledgment of the importance of discussing and reporting assaults. Still according to the findings, sexual violence affects adolescents from different socioeconomic and educational contexts. Therefore, it is essential to identify factors that increase their vulnerability^7^ ^,^ ^8^.

Adolescents who were assaulted and bullied presented a higher prevalence of SV. On the one hand, adolescents exposed to violence have a higher risk of developing aggressive and antisocial behaviors, considering that living with peers who present risk behaviors favors the adoption of such practices^48^ ^,^ ^49^. On the other hand, the perception of positive relationships with peers has been linked to a lower involvement in risk behaviors^50^ ^,^ ^51^.

The perception of insecurity was also associated with SV. Adolescents who reported feeling unsafe getting to and from school and at school presented higher prevalence of SV, as also identified in Brazilian studies on adolescents^11^ ^,^ ^27^. This insecurity can be associated with the lack of effective public policies, such as quality public transport, safety, and urban infrastructure, increasing the occurrences of violence such as robberies, assaults, and conflicts in public spaces^52^.

Conversely, some protective factors were also identified. Lower prevalence of SV was observed among adolescents whose parents demonstrated understanding their issues and concerns. This corroborates previous studies conducted in Brazil, whose authors showed that parental concern benefits the health of adolescents and reduces their exposure to risk factors for SV^53^ ^,^ ^54^ ^,^ ^55^.

Adolescents who attended public schools had a lower prevalence of SV, a result that differs from that observed in the study on 2015 PeNSE^27^. Although these students face socioeconomic challenges, community support networks, such as social services, may consist in protective factors^56^ ^,^ ^57^. The relationship between type of school and violence goes beyond the socioeconomic aspect, involving an interaction of family, social, and cultural factors that should to be deepened.

Among the limitations of this study, the cross-sectional design of PeNSE stands out, which makes it impossible to establish causal relationships between the analyzed factors. In addition, the research represents only adolescents who attend school, not being generalizable to all Brazilian adolescents. It should be noted that the 2019 edition of PeNSE expanded the diversity of the sample by including schools located in Indigenous areas and of difficult access, which contributes to the greater representativeness of the school population, although it does not minimize the methodological limitations of the study. Another limitation is the use of self-administered questionnaires, subject to biases that can underestimate or overestimate the data. Furthermore, the PeNSE violence module has not undergone formal validation, which may affect the accuracy and comparability of data. The interpretation of the questions can also vary among the respondents, compromising the uniformity of the answers. Nonetheless, PeNSE has adopted recognized international methodologies — such as the Global School-Based Student Health Survey, the Health Behaviour in School-Aged Children, and the Youth Risk Behavior Surveillance System — whose instruments are validated and reliable. Thus, the results strongly reflect the reality of adolescents aged 13 to 17 years.

We conclude that the prevalence of SV among adolescent girls was 22.2% and was associated with individual, social, and community factors. Higher PR values were verified among students aged 16 to 17 years who used illicit drugs, consumed alcohol, have had sexual relations, reported feelings of sadness, believed that life was not worth living, had mothers with higher levels of education, were bullied, were assaulted by friends, and felt unsafe getting to and from school and at school. Our findings evidence the complexity of the issue and the need for integrated interventions and intersectoral public policies that promote safer environments for adolescents.
