# Supplementary material for: Prevalence and factors associated with sexual violence against adolescent girls in Brazil: results of the 2019 National Survey of School Health
Source: Rev Bras Epidemiol. 2026 Apr 3;29:e260016. doi: 10.1590/1980-549720260016 (PMC13053035; doi:10.1590/1980-549720260016)
Supplement: Translation

# Translation: Prevalência e fatores associados à violência sexual contra adolescentes do sexo feminino no Brasil: resultados da Pesquisa Nacional da Saúde do Escolar, 2019

**DOI:** 10.1590/1980-549720260016.2

**Authors:** Maria Luiza Sady Prates Pinheiro, Alanna Gomes da Silva, Nádia Machado de Vasconcelos, Deborah Carvalho Malta

**Affiliations:** I Universidade Federal de Minas Gerais, Escola de Enfermagem. Departamento de Enfermagem Materno-infantil e Saúde Pública - Belo Horizonte (MG), Brasil.; II Universidade Federal de Minas Gerais, Faculdade de Medicina. Departamento de Medicina Preventiva e Social - Belo Horizonte (MG), Brasil.; III Universidade Federal de Minas Gerais, Programa de Pós-Graduação em Saúde Pública - Belo Horizonte (MG), Brasil.

**Keywords:** Epidemiologia, Saúde do adolescente, Inquéritos epidemiológicos, Brasil, Delitos sexuais

## Abstract

**Objetivo:**

Analisar a prevalência de violência sexual e fatores associados entre meninas adolescentes de 13 a 17 anos no Brasil, considerando aspectos individuais, relacionais e comunitários.

**Métodos:**

Estudo transversal com dados da Pesquisa Nacional de Saúde do Escolar (PeNSE) de 2019. Foram realizadas análises descritivas e foram calculadas as Razões de Prevalência (RP) com intervalos de confiança de 95%.

**Resultados:**

A prevalência de violência sexual foi de 22,2% (IC95% 21,5-23), sendo o principal agressor um desconhecido (22,5%; IC95% 21,2-23,8). Maiores prevalências foram observadas entre adolescentes de 16 a 17 anos (RP 1,2; IC95% 1,1-1,3), que usaram drogas (RP 1,3; IC95% 1,1-1,4), consumiram álcool (RP 1,2; IC95% 1,1-1,3), com histórico de vida sexual (RP 1,5; IC95% 1,4-1,7), que relataram tristeza (RP 1,9; IC95% 1,4-2,5), que tinham mães com ensino superior (RP 1,2; IC95% 1,1-1,3), que sofreram bullying (RP 1,2; IC95% 1,2-1,3), agressões por amigos (RP 1,2; IC95% 1,1-1,4), que tiveram a sensação de insegurança no trajeto casa-escola (RP 1,2; IC95% 1,1-1,4) e na escola (RP 1,3; IC95% 1,1-1,4).

**Conclusão:**

A violência sexual em meninas apresentou elevada prevalência entre as escolares de 16 a 17 anos no Brasil, além de estar associada a fatores individuais, sociais e comunitários. São necessárias intervenções multifacetadas, bem como o fortalecimento de políticas públicas intersetoriais e estratégias de proteção para garantir um ambiente mais seguro.

## INTRODUÇÃO

A Violência Sexual (VS) contra meninas adolescentes é uma grave violação dos direitos humanos e um persistente problema de saúde pública^1^. Trata-se de qualquer ação de caráter sexual, concretizada ou tentada, incluindo exploração sexual, que cause danos físicos, psicológicos ou sociais, bem como condutas que obriguem a vítima a presenciar ou praticar atos sexuais, de forma presencial ou virtual^2^ ^,^ ^3^. Essa forma de violência é prevalente nessa faixa etária^2^ ^,^ ^4^ ^,^ ^5^, afetando o desenvolvimento, a autoestima, a saúde mental e o desempenho escolar das adolescentes^6^.

A violência é um fenômeno complexo, relacionado a fatores culturais, sociais e econômicos^7^ ^,^ ^8^, afetando especialmente grupos vulneráveis, como crianças, adolescentes, meninas e mulheres^9^ ^,^ ^10^. Nesse contexto, a VS contra meninas adolescentes reflete uma dinâmica estrutural de desigualdades e violações, sendo reflexo de uma sociedade patriarcal que sustenta a dominação masculina em diversas esferas^11^ ^,^ ^12^ ^,^ ^13^.

A adolescência é marcada por mudanças biológicas, psicológicas e emocionais essenciais ao desenvolvimento humano^7^ ^,^ ^14^. Nesse período, o contexto familiar exerce grande influência no bem-estar das adolescentes, tornando-as mais vulneráveis às dinâmicas físicas e sociais do ambiente^15^. Em situações de vulnerabilidade social, escolar e econômica, esses fatores se intensificam, ampliando o risco de exposição à violência e a outros agravos à saúde^16^ ^,^ ^17^.

A violência é uma das principais causas de morbimortalidade entre adolescentes no Brasil^18^, com impactos duradouros em várias áreas da vida^7^ ^,^ ^14^ ^,^ ^19^. Está associada ao maior risco de transtornos mentais, ao uso de álcool, drogas, tabaco e práticas sexuais desprotegidas^20^. Também aumenta a exposição a doenças crônicas, como câncer, diabetes e cardiovasculares, além de infecções, como o HIV^19^.

Globalmente, estima-se que 370 milhões de meninas e mulheres tenham sofrido estupro ou abuso sexual durante a infância^21^. De 2015 a 2021, 92,7% das notificações de VS suspeita ou confirmada foram em meninas adolescentes^22^. Em 2022, 87,7% das vítimas de VS com menos de 19 anos eram do sexo feminino^23^, e, em 2023, 49,6% das agressões registradas contra adolescentes de 10 a 14 anos foram de natureza sexual^2^. Ainda em 2023, os casos de estupro cresceram 1,54% em relação ao ano anterior, sendo a maioria das vítimas mulheres^24^.

Devido ao impacto e à magnitude da violência de gênero, o Brasil está comprometido com a Agenda 2030 dos Objetivos do Desenvolvimento Sustentável (ODS), que busca alcançar a igualdade de gênero e o empoderamento de mulheres e meninas, incluindo a eliminação da violência de gênero^25^.

No Brasil, ainda são limitados os estudos que investigam de forma abrangente os fatores associados à VS em meninas adolescentes com base em dados populacionais nacionais. A maioria das pesquisas foca em amostras locais em ambos os sexos^26^ ^,^ ^27^ ^,^ ^28^ ^,^ ^29^ ou trata a violência de forma geral^4^ ^,^ ^8^ ^,^ ^11^ ^,^ ^30^ ^,^ ^31^. Apesar de avançarem na compreensão da vulnerabilidade e dos fatores de risco da violência sexual, esses estudos têm limitações metodológicas, como amostras pequenas, foco regional e falta de análises por sexo. Além disso, poucos abordam de maneira integrada dimensões relacionais e comunitárias. Ao utilizar dados da Pesquisa Nacional de Saúde do Escolar (PeNSE) 2019, esta análise contribui para ampliar o conhecimento sobre a VS entre meninas em escala nacional, oferecendo subsídios para políticas públicas mais eficazes. Diferentemente de estudos da edição de 2015^27^ ^,^ ^32^, que apresentou dados agregados por sexo, a de 2019 permite uma análise mais sensível às desigualdades de gênero.

Diante disso, o objetivo deste estudo foi analisar a prevalência de VS e fatores associados entre meninas adolescentes de 13 a 17 anos no Brasil, considerando aspectos individuais, relacionais e comunitários.

## MÉTODOS

Estudo transversal e analítico, com dados da PeNSE 2019, realizado pelo Instituto Brasileiro de Geografia e Estatística (IBGE) em parceria com o Ministério da Saúde^33^.

O plano amostral da pesquisa foi constituído por conglomerados em dois estágios: escolas; e turmas de alunos matriculados, e considerou os seguintes níveis geográficos: Brasil; grandes regiões; unidades da federação; municípios das capitais; e Distrito Federal^33^.

Os dados foram coletados via questionário estruturado autoaplicável, preenchido pelos estudantes em um *smartphone*. Os pesos de escolas, turmas e alunos foram ajustados a partir dos dados do Censo Escolar. Em 2019, foram analisados 125.123 questionários, coletados em 4.242 escolas e em 6.612 turmas, com participação de 189.857 alunos matriculados e de 183.264 alunos frequentes^33^.

Os participantes da PeNSE são adolescentes de 13 a 17 anos, matriculados e frequentes no 7^o^ ao 9^o^ ano do Ensino Fundamental e na 1^a^ a 3^a^ série do Ensino Médio em escolas públicas e privadas do Brasil^33^. Para esse estudo, considerou-se a população de adolescentes do sexo feminino, entre 13 e 17 anos, que tiveram seus questionários válidos (n=63.148).

A variável dependente do estudo foi a VS, sendo construída a partir dos indicadores presentes na seção “Segurança” do questionário, referindo-se a situações de segurança no ambiente em que o escolar vive. A variável VS é composta pelas adolescentes do sexo feminino que responderam “sim” para um dos seguintes indicadores^26^:

1. Abuso sexual: prevalência de meninas escolares que, alguma vez na vida, foram tocadas por alguém, manipuladas, beijadas ou que sofreram exposição de partes do corpo contra a sua vontade;
2. Estupro: prevalência de meninas escolares que, alguma vez na vida, foram ameaçadas por alguém, intimidadas ou obrigadas a ter relações sexuais ou qualquer outro ato sexual contra a sua vontade.

Para as meninas que responderam “sim” em um dos indicadores, foram analisadas também as variáveis relacionadas: idade do primeiro estupro e identidade do agressor. A idade foi investigada por meio da pergunta: “Que idade você tinha quando alguém ameaçou, intimidou ou obrigou a ter relações sexuais ou qualquer outro ato sexual contra a sua vontade pela primeira vez?” (9 anos ou menos; 10 a 13 anos; 14 a 15 anos; e 16 a 17 anos). Já a identidade do agressor foi classificada nas seguintes categorias: namorado(a), ex-namorado(a), ficante, *crush*; amigo(a); pai, mãe, padrasto ou madrasta, outros familiares, desconhecido(a) e outros.

As variáveis independentes relacionadas à VS foram construídas com base no modelo ecológico da violência, conforme proposto por Dahlberg e Krug^34^, o qual, por sua vez, foi adaptado do modelo ecológico do desenvolvimento humano de Bronfenbrenner^35^. O modelo avalia os fatores relacionados à violência em quatro níveis: indivíduo, relações, comunidade e sociedade. O primeiro nível está relacionado às características individuais; o segundo, às relações sociais próximas; o terceiro, aos contextos comunitários em que o indivíduo está inserido; e o quarto, aos fatores sociais mais amplos que afetam as taxas de violência^34^. Neste estudo, o modelo foi utilizado exclusivamente como referencial teórico para a categorização das variáveis e interpretação dos resultados, sem aplicação de técnicas estatísticas de análise multinível. Diante disso, as variáveis foram divididas em três níveis: indivíduo, relações e comunidade. O quarto nível, sociedade, não foi avaliado, pois a PeNSE não abrange esse aspecto.

As variáveis independentes incluídas no modelo foram descritas por meio de frequências relativas (%) e pelos respectivos Intervalos de Confiança de 95% (IC95%), com base nas categorias disponíveis na base de dados. A seguir, apresenta-se a definição e a categorização dessas variáveis, segundo o modelo ecológico da violência adotado para a análise:

• Fatores individuais (nível 1): faixa etária (13 a 15 anos; 16 e 17 anos); cor da pele (branca, preta, parda e outras — amarela e indígena); uso de drogas ilícitas — maconha e crack (sim; não); consumo de bebidas alcoólicas nos últimos 30 dias (sim; não); uso de cigarros nos últimos 30 dias (sim; não); uso de outros produtos do tabaco nos últimos 30 dias (sim; não); histórico de atividade sexual (sim; não); sentimento de tristeza (sim; não); e sentimento de que a vida não vale a pena ser vivida (sim; não).
• Relações sociais próximas (nível 2): escolaridade materna (sem escolaridade ou fundamental completo e incompleto, médio completo e incompleto ou superior incompleto e superior completo); pais ou responsáveis fumantes (sim; não); pais ou responsáveis consomem bebidas alcoólicas (sim; não); moram com os pais (sim; não); realizam refeição com os pais (sim; não); os pais entendem seus problemas e preocupações (sim; não); tem amigos próximos (sim; não); teve envolvimento em brigas (sim; não); pratica e sofre *bullying* (sim; não); foi agredido por amigos (sim; não); e tem amigos prestativos (sim; não).
• Contextos comunitários (nível 3): situação de moradia (urbano e rural); dependência administrativa da escola (privada e pública); segurança no caminho entre casa e escola (sim; não); segurança na escola (sim; não); e região (Nordeste, Norte, Sudeste, Sul e Centro-Oeste).

Foram realizadas análises descritivas, por meio do cálculo das prevalências e dos respectivos Intervalos de Confiança de 95% (IC95%) das variáveis dependentes e independentes, bem como da prevalência da violência por Unidade Federativa. Calcularam-se as Razões de Prevalência (RP) com seus respectivos IC95%, utilizando modelos de regressão de Poisson com variância robusta. Realizaram-se análises bivariadas para se obterem as RP brutas e os IC95%. Procedeu-se à análise multivariável, sendo incluídas no modelo as variáveis com p≤0,05 nas análises brutas, e utilizou-se o método *backward* para a construção do modelo de regressão multivariável. No modelo final, consideraram-se como fatores associados as variáveis com p≤0,05. A estrutura do processo de amostragem e os pesos de pós-estratificação foram considerados na análise dos resultados. Utilizou-se o *software for Statistics and Data Science* (*Stata)*, versão 14.0, por meio do módulo *survey*, que considera efeitos do plano amostral.

A PeNSE 2019 está em conformidade com as Diretrizes e Normas Regulamentadoras de Pesquisa Envolvendo Seres Humanos e foi aprovada pela Comissão Nacional de Ética em Pesquisa do Ministério da Saúde, sob parecer n^o^ 3.249.268 em 08 de abril de 2019. Os escolares foram informados sobre a pesquisa, sobre sua livre participação e que poderiam desistir, caso não se sentissem à vontade para responder às questões.

## Declaração de Disponibilidade de Dados:

Todo o conjunto de dados que dá suporte aos resultados deste estudo foi publicado no próprio artigo.

## RESULTADOS

O abuso sexual ocorreu em 20,2% (IC95% 19,5-21), o estupro em 8,9% (IC95% 8,4-9,3) e a VS, definida como a ocorrência de abuso sexual e/ou estupro, foi relatada por 22,2% (IC95% 21,5-23) dessas adolescentes. A coocorrência de abuso sexual e estupro foi observada em 7,0% (IC95% 6,6-7,4) das participantes. Os principais responsáveis pela VS foram desconhecidos (22,5%; IC95% 21,2-23,8), seguidos por um familiar (19,1%; IC95% 17,7-20,5), namorado (19,0%; IC95% 17,7-20,4), amigo (16,9%; IC95% 15,7-18,2), outros (16,1%; IC95% 14,8-17,6) e pais (6,6%; IC95% 5,8-7,5).

No que diz respeito aos fatores individuais, 27,1% (IC95% 26,0-28,3) das adolescentes que relataram VS estavam na faixa etária de 16 a 17 anos. No entanto, 37,3% afirmaram que o primeiro episódio de violência ocorreu entre os 10 e 13 anos — 30,4% com 9 anos ou menos; 24,5% entre 14 e 15 anos; e 7,9% entre 16 e 17 anos. A VS foi mais prevalente entre as meninas que relataram uso de drogas ilícitas (RP 46,2%; IC95% 42,4-50,1); que consumiram bebidas alcoólicas nos últimos 30 dias (RP 31,6%; IC95% 30,2-33,1); que fumaram cigarro nos últimos 30 dias (RP 40,7%; IC95% 36,6-44,8); e que relataram ter histórico de atividade sexual (RP 34,0%; IC95% 32,7-35,3), conforme Tabela 1.

**Tabela 1.** Características e Razão de Prevalência bruta da violência sexual das adolescentes do sexo feminino que sofreram violência sexual. Pesquisa Nacional de Saúde do Escolar, 2019.

Variáveis	% (IC95%)	RPb (IC95%)	p-valor
Fatores individuais			
Faixa etária (anos)			
13 a 15	19,6 (18,7-20,5)	*	<0,001
16 e 17	27,1 (26,0-28,3)	1,4 (1,3-1,5)	
Cor da pele			
Branca	23,8 (22,6-25)	*	<0,001
Preta	21,8 (19,9-23,8)	0,9 (0,8-1)	
Parda	20,9 (20-21,9)	0,9 (0,8-0,9)	
Outras	24,4 (22-27)	1,0 (0,9-1,1)	
Uso de drogas ilícitas			
Não	21,0 (20,3-21,8)	*	<0,001
Sim	46,2 (42,4-50,1)	2,2 (2-2,4)	
Consumo de bebidas alcoólicas nos últimos 30 dias			
Não	18,2 (17,5-18,9)	*	<0,001
Sim	31,6 (30,2-33,1)	1,7 (1,6-1,8)	
Uso de cigarros nos últimos 30 dias			
Não	21,0 (20,2-21,7)	*	<0,001
Sim	40,7 (36,6-44,8)	1,9 (1,7-2,2)	
Uso de outros produtos do tabaco nos últimos 30 dias			
Não	20,3 (19,5-21,1)	*	<0,001
Sim	36,8 (34,2-39,4)	1,8 (1,7-2)	
Histórico de atividade sexual			
Não	17,0 (16,2-17,8)	*	<0,001
Sim	34,0 (32,7-35,3)	2,0 (1,9-2,1)	
Sentimento de tristeza			
Não	7,8 (6-10)	*	<0,001
Sim	23,0 (22,3-23,8)	3,0 (2,3-3,8)	
Sentimento de que a vida não vale a pena ser vivida			
Não	12,8 (11,8-13,8	*	<0,001
Sim	27,4 (26,4-28,3)	2,1 (2-2,3)	
Relações sociais próximas			
Escolaridade materna			
Sem escolaridade e fundamental c/i	21,2 (19,9-22,5)	*	<0,001
Ensino médio c/i e superior incompleto	24,1 (22,9-25,3)	1,1 (1-1,2)	
Ensino superior completo	25,8 (24,3-27,3)	1,2 (1,1-1,3)	
Pais ou responsáveis fumantes			
Não	21,2 (20,4-22,1)	*	<0,001
Sim	24,8 (23,4-26,2)	1,2 (1,1-1,2)	
Pais ou responsáveis consomem bebidas alcóolicas			
Não	19,0 (17,9-20,1)	*	<0,001
Sim	24,2 (23,2-25,2)	1,3 (1,2-1,4)	
Moram com os pais			
Não	26,8 (24,6-29,2)	*	<0,001
Sim	21,9 (21,1-22,7)	0,8 (0,7-0,9)	
Realizam refeição com os pais			
Não	34,4 (31,5-37,5)	*	<0,001
Sim	21,3 (20,5-22,1)	0,6 (0,6-0,7)	
Pais entendem seus problemas e preocupações			
Não	31,3 (29,6-33,1)	*	<0,001
Sim	20,2 (19,4-21)	0,6 (0,6-0,7)	
Tem amigos próximos			
Não	30,5 (26,7-34,5)	*	<0,001
Sim	21,9 (21,2-22,7)	0,7 (0,6-0,8)	
Envolvimento em brigas			
Não	21,3 (20,5-22,1)	*	<0,001
Sim	35,8 (33,1-38,6)	1,7 (1,5-1,8)	
Prática de *bullying*			
Não	21,1 (20,4-21,9)	*	<0,001
Sim	33,1 (30,8-35,6)	1,6 (1,5-1,7)	
Ter sofrido *bullying*			
Não	17,4 (16,6-18,2)	*	<0,001
Sim	28,6 (27,4-29,8)	1,6 (1,6-1,7)	
Agredido por amigos			
Não	20,7 (19,9-21,4)	*	<0,001
Sim	34,4 (32-36,9)	1,7 (1,5-1,8)	
Tem amigos prestativos			
Não	22,1 (19,4-25,2)	*	0,090
Sim	22,3 (21,5-23)	1,0 (0,9-1,1)	
Contextos comunitários			
Situação de moradia			
Urbano	22,7 (21,9-23,4)	*	0,001
Rural	17,3 (14,5-20,5)	0,8 (0,6-0,9)	
Dependência administrativa da escola			
Privado	24,9 (24-25,8)	*	<0,001
Público	21,8 (21-22,7)	0,9 (0,8-0,9)	
Segurança no caminho entre casa e escola			
Sim	20,4 (19,7-21,1)	*	<0,001
Não	34,4 (31,7-37,2)	1,7 (1,6-1,8)	
Segurança na escola			
Sim	20,1 (19,4-20,9)	*	<0,001
Não	36,3 (34,0-38,7)	1,8 (1,7-1,9)	
Região			
Norte	23,2 (21,1-25,5)	*	0,012
Nordeste	20,3 (19,4-21,3)	0,9 (0,8-1)	
Sudeste	23,3 (21,7-24,9)	1,0 (0,9-1,1)	
Sul	22,2 (20,4-24,2)	1,0 (0,8-1,1)	
Centro-Oeste	23,0 (21,6-24,5)	1,0 (0,9-1,1)	

IC95%: intervalo de confiança de 95%; RPb: razão de prevalência bruta; c/i: completo/incompleto.

*Variável de referência da razão de prevalência.

Quanto às relações sociais próximas, teve maior prevalência entre as meninas que tinham pais fumantes (RP 24,8%; IC95% 23,4-26,2); pais que consumiam bebidas alcoólicas (RP 24,2%; IC95% 23,2-25,2); que não moravam com os pais (RP 26,8%; IC95% 24,6-29,2); e que não faziam refeição com os pais (RP 34,4%; IC95% 31,5-37,5), conforme Tabela 1.

Com relação aos contextos comunitários, tiveram maiores prevalências entre aquelas que moravam em região urbana (RP 22,7%; IC95% 21,9-23,4); que estudavam em escolas privadas (RP 24,9%; IC95% 24-25,8); que não se sentiam seguras no caminho de casa para a escola (RP 34,4%; IC95% 31,7-37,2); e na escola (RP 36,3%; IC95% 34,0-38,7), de acordo com dados da Tabela 1.

O Gráfico 1 apresenta as prevalências de adolescentes que sofreram VS, de acordo com as unidades da federação. Embora o Amapá (27,7%) apresente a maior prevalência, seguido por Amazonas (25,4%) e Mato Grosso do Sul (24,2%), os intervalos de confiança das estimativas se sobrepõem, o que implica que as diferenças observadas podem não ser estatisticamente significativas.

**Gráfico 1.** Prevalência de violência sexual em adolescentes do sexo feminino de 13 a 17 anos, segundo Unidades da Federação. Pesquisa Nacional de Saúde do Escolar, 2019.

Na Tabela 2, foi apresentado o modelo multivariado final. A maior RP de VS foi observada entre adolescentes de 16 a 17 anos (RP 1,17; IC95% 1,08-1,27); que fizeram uso de drogas (RP 1,27; IC95% 1,14-1,41); consumiram álcool nos últimos 30 dias (RP 1,18; IC95% 1,10-1,27); com histórico de atividade sexual (RP 1,54; IC95% 1,42-1,67); que relataram tristeza (RP: 1,85; IC95%: 1,35-2,54) e acreditaram que a vida não valia a pena ser vivida (RP 1,51; IC95% 1,38-1,66). Além disso, ter mães com ensino superior completo (RP 1,21; IC95% 1,09-1,34), sofrer *bullying* (RP 1,24; IC95% 1,16-1,33), ter sido agredida por amigos (RP 1,24; IC95% 1,14-1,35), sentir-se insegura no trajeto entre casa e escola (RP 1,24; IC95% 1,14-1,36) e na escola (RP 1,25; IC95% 1,14-1,36) também apresentaram associações positivas. Por outro lado, menores Razões de Prevalência (RP) de VS foram verificadas entre as adolescentes que tinham pais que compreendiam seus problemas e suas preocupações (RP 0,83; IC95% 0,74-0,93) e entre as que estudavam em escolas públicas (RP 0,84; IC95% 0,78-0,89).

**Tabela 2.** Modelo multivariado da violência sexual em adolescentes do sexo feminino de 13 a 17 anos. Pesquisa Nacional de Saúde do Escolar, Brasil, 2019.

Variáveis	RPaj (IC95%)	p-valor
Fatores individuais		
Faixa etária (anos)		
13 a 15	*	<0,001
16 e 17	1,17 (1,08-1,27)	
Cor da pele		
Branca	*	
Preta	0,88 (0,79-0,98)	0,031
Parda	0,94 (0,87-1,01)	0,121
Outras	1,03 (0,92-1,16)	0,542
Uso de drogas ilícitas		
Não	*	<0,001
Sim	1,27 (1,14-1,41)	
Consumo de bebidas alcoólicas nos últimos 30 dias		
Não	*	<0,001
Sim	1,18 (1,10-1,27)	
Histórico de atividade sexual		
Não	*	<0,001
Sim	1,54 (1,42-1,67)	
Sentimento de tristeza		
Não	*	<0,001
Sim	1,85 (1,35-2,54)	
Sentimento de que a vida não vale a pena ser vivida		
Não	*	<0,001
Sim	1,51 (1,38-1,66)	
Relações sociais próximas		
Escolaridade materna		
Sem escolaridade e fundamental completo e incompleto	*	<0,001
Ensino médio completo e incompleto e superior incompleto	1,14 (1,06-1,22)	
Ensino superior completo	1,21 (1,09-1,34)	
Realizam refeição com os pais		
Não	*	0,001
Sim	0,83 (0,74-0,93)	
Pais entendem seus problemas e preocupações		
Não	*	<0,001
Sim	0,83 (0,74-0,93)	
Prática de *bullying*		
Não	*	0,031
Sim	1,10 (1,00-1,19)	
Ter sofrido *bullying*		
Não	*	<0,001
Sim	1,24 (1,16-1,33)	
Agredido por amigos		
Não	*	<0,001
Sim	1,24 (1,14-1,35)	
Contextos comunitários		
Dependência administrativa da escola		
Privado	*	<0,001
Público	0,84 (0,78-0,89)	
Segurança no caminho entre casa e escola		
Sim	*	<0,001
Não	1,24 (1,14-1,36)	
Segurança na escola		
Sim	*	<0,001
Não	1,25 (1,14-1,36)	
Região		
Norte	*	
Nordeste	0,88 (0,78-0,98)	0,033
Sudeste	0,86 (0,76-0,98)	0,028
Sul	0,89 (0,77-1,02)	0,107
Centro-Oeste	0,90 (0,80-1,02)	0,118

RPaj: razão de prevalência ajustada; IC95%: intervalo de confiança de 95%.

*Variável de referência da razão de prevalência.

## DISCUSSÃO

Cerca de 22,2% das adolescentes brasileiras do sexo feminino foram vítimas de VS. As maiores RPs foram entre as adolescentes de 16 e 17 anos; que usavam drogas ilícitas e álcool; que tiveram relações sexuais; que relataram sentimentos de tristeza e de que a vida não vale a pena ser vivida; que tinham mães com o ensino superior completo; que sofreram *bullying*; que foram agredidas pelos amigos; e que sentiam insegurança no caminho de casa para a escola e dentro da escola. Por outro lado, as menores RPs foram observadas entre as que estudavam em escolas públicas e entre aquelas cujos pais entendiam seus problemas e preocupações.

A prevalência de VS observada neste estudo foi consideravelmente superior à encontrada em uma pesquisa que utilizou dados da PeNSE de 2015, a qual apontou uma taxa de 4,3% entre meninas do 9^o^ ano do Ensino Fundamental^27^. Essa discrepância pode ser parcialmente explicada por diferenças metodológicas relevantes entre as duas edições da pesquisa. Em 2015, a pergunta sobre VS era formulada de forma mais genérica e sem exemplificações, o que pode ter limitado o reconhecimento da vivência de situações de violência por parte das adolescentes. Já em 2019, além da ampliação da população-alvo (7^o^ ano do ensino fundamental ao 3^o^ ano do ensino médio), houve uma diferenciação entre abuso sexual e estupro, com perguntas mais específicas e exemplificativas, favorecendo maior compreensão e relato.

Embora os relatos de VS tenham predominado entre as adolescentes de 16 a 17 anos, possivelmente devido ao maior tempo de vida e à maior compreensão da violência, observou-se maior prevalência do primeiro episódio de VS na infância e na pré-adolescência, entre 10 e 13 anos. Outros estudos apontam meninas de 10 a 14 anos como o grupo mais vulnerável à VS, frequentemente ocorrida no ambiente doméstico^2^ ^,^ ^27^.

Globalmente, estima-se que 1 em cada 5 mulheres tenha sido vítima de VS durante a infância^36^. Fatores como baixa escolaridade, acesso limitado a meios de proteção e dependência econômica, especialmente quando não acompanhada de suporte familiar adequado, aumentam a vulnerabilidade de meninas adolescentes à VS^37^.

No que diz respeito aos agressores, os principais foram indivíduos desconhecidos, seguidos por familiares. No entanto, estudos apontam a residência como o principal cenário de ocorrência dessa violência, sendo os familiares os principais agressores^11^ ^,^ ^21^ ^,^ ^38^. Essa discrepância pode estar associada à maior capacidade das vítimas de identificar e relatar episódios de violência ocorridos fora do ambiente familiar, enquanto a naturalização da violência no contexto doméstico, influenciada por fatores socioculturais, pode dificultar seu reconhecimento e sua denúncia^39^.

Em relação ao consumo de substâncias, as adolescentes que consumiram drogas ilícitas apresentaram maior prevalência de VS. Nessa fase, a busca por meios para lidar com experiências difíceis as expõe ao consumo de drogas para enfrentamento^40^ ^,^ ^41^ ^,^ ^42^. Estudos mostram que o uso desse tipo de substância, tanto das lícitas quanto das ilícitas, é um comportamento que aumenta a vulnerabilidade do adolescente, provocando alterações no seu comportamento, como a diminuição da capacidade de percepção do ambiente, tornando a adolescente mais vulnerável^27^ ^,^ ^28^ ^,^ ^31^ ^,^ ^42^.

Outro fator relevante é que meninas que já tiveram relação sexual apresentaram maior prevalência de VS. A adolescência, marcada por descobertas e pela maior interação social, inclui relações afetivas e sexuais^14^, que podem aumentar a exposição a vulnerabilidades. Assim, adolescentes sexualmente ativas tendem a enfrentar mais situações de risco relacionadas à VS^43^ ^,^ ^44^. Os comportamentos sexuais de risco, como maior número de parceiros e maior exposição a infecções sexualmente transmissíveis, podem gerar impactos negativos no desenvolvimento físico, mental e psicossocial, com repercussões significativas na vida adulta^45^.

Além disso, as adolescentes que apresentaram sentimentos de tristeza e a percepção de que a vida não vale a pena tiveram maior prevalência de VS. A exposição à violência na adolescência pode estar associada a um risco aproximadamente duas vezes maior de desenvolver transtornos de humor e ansiedade^46^. Além disso, pode aumentar as tentativas de suicídio, internações psiquiátricas, automutilação, transtornos de humor e comportamentais, sintomas dissociativos, transtorno de estresse pós-traumático e somatizações^47^.

Em relação à VS, as adolescentes que tinham mães com maior escolaridade tiveram maior prevalência. No entanto, um estudo com dados da PeNSE 2015 identificou maior prevalência de VS entre adolescentes cujas mães tinham menor escolaridade^27^. A maior escolaridade pode facilitar a identificação de casos de violência e o reconhecimento da importância de discutir e denunciar agressões. Os achados também indicam que a violência sexual atinge adolescentes de diferentes contextos socioeconômicos e educacionais. Por isso, é essencial identificar fatores que aumentam sua vulnerabilidade^7^ ^,^ ^8^.

As adolescentes que foram agredidas e sofreram *bullying* apresentaram maior prevalência de VS. Adolescentes expostos à violência têm maior risco de desenvolver comportamentos agressivos e antissociais, pois conviver com pares que apresentam comportamentos de risco favorece a adoção dessas práticas^48^ ^,^ ^49^. Por outro lado, a percepção de relacionamentos positivos com os pares tem sido vinculada a um menor envolvimento em comportamentos de risco^50^ ^,^ ^51^.

A percepção de insegurança também se mostrou associada à VS. As adolescentes que relataram insegurança no trajeto de casa para a escola e na escola apresentaram maior prevalência de VS, conforme também identificado em estudos brasileiros com adolescentes^11^ ^,^ ^27^. Essa insegurança pode ser associada à falta de políticas públicas eficazes, como transporte público de qualidade, segurança e infraestrutura urbana, aumentando as ocorrências de violência, como assaltos, agressões e conflitos nos espaços públicos^52^.

Por outro lado, alguns fatores protetores também foram identificados. As menores prevalências de VS foram observadas entre as adolescentes cujos pais demonstraram compreensão em relação aos seus problemas e preocupações. Corroborando com pesquisas anteriores no Brasil, que mostram que a preocupação parental beneficia a saúde das adolescentes e reduz sua exposição a fatores de risco para a VS^53^ ^,^ ^54^ ^,^ ^55^.

As adolescentes que estudavam em escolas públicas apresentaram menor prevalência de VS, resultado que difere do observado no estudo com a PeNSE 2015^27^. Embora essas estudantes enfrentem desafios socioeconômicos, redes de apoio comunitário, como serviços sociais, podem funcionar como fator protetor^56^ ^,^ ^57^. A relação entre o tipo de escola e a violência vai além do aspecto socioeconômico, envolvendo uma interação de fatores familiares, sociais e culturais que precisam ser aprofundados.

Entre as limitações deste estudo, destaca-se o delineamento transversal da PeNSE, o que impossibilita estabelecer relações causais entre os fatores analisados. Além disso, a pesquisa representa apenas adolescentes que frequentam a escola, não sendo generalizável a todos os adolescentes brasileiros. Cabe destacar que a edição de 2019 da PeNSE ampliou a diversidade da amostra ao incluir escolas localizadas em áreas indígenas e de difícil acesso, o que contribui para a maior representatividade da população escolar, embora não minimize as limitações metodológicas do estudo. Outra limitação é o uso de questionários autorreferidos, sujeitos a vieses que podem sub ou superestimar os dados. Ademais, o módulo de violência da PeNSE não passou por validação formal, o que pode afetar a precisão e a comparabilidade dos dados. A interpretação das perguntas também pode variar entre os respondentes, comprometendo a uniformidade das respostas. Apesar disso, a PeNSE adotou metodologias internacionais reconhecidas, como o *Global School-Based Student Health Survey*, o *Health Behaviour in School-Aged Children* e o *Youth Risk Behavior Surveillance System*, cujos instrumentos são validados e confiáveis. Assim, os resultados refletem de forma robusta a realidade de adolescentes escolarizados entre 13 e 17 anos.

Conclui-se que a prevalência de VS entre adolescentes do sexo feminino foi de 22,2% e esteve associada a fatores individuais, sociais e comunitários. As maiores razões de prevalência ocorreram entre estudantes de 16 a 17 anos que usavam drogas ilícitas, consumiam álcool, tinham iniciado a vida sexual, relataram sentimentos de tristeza, acreditavam que a vida não valia a pena ser vivida, tinham mães com maior escolaridade, sofriam *bullying*, agressões de amigos e sentiam-se inseguras no trajeto e no ambiente escolar. Os resultados evidenciam a complexidade do problema e a necessidade de intervenções integradas e políticas públicas intersetoriais que promovam ambientes mais seguros para as adolescentes.
